# Risk of Thromboembolism in Non-Valvular Atrial Fibrillation With or Without Clinical Hyperthyroidism

**DOI:** 10.5334/gh.871

**Published:** 2021-06-17

**Authors:** Yu-Sheng Lin, Hsin-Yu Tsai, Chia-Ying Lin, Victor Chien-Chia Wu, Tien-Hsing Chen, Teng-Yao Yang, Victor Aboyans, Mien-Cheng Chen

**Affiliations:** 1 Division of Cardiology, Department of Internal Medicine, Chang Gung Memorial Hospital, Chiayi, TW; 2 Graduate Institute of Clinical Medical Sciences, College of Medicine, Chang Gung University, TW; 3 Department of Internal Medicine, Chang Gung Memorial Hospital, Linkou Medical Center, Taoyuan City, TW; 4 Division of Cardiology, Chang Gung Memorial Hospital, Linkou Medical Center, Taoyuan City, TW; 5 Division of Cardiology, Department of Internal Medicine, Chang Gung Memorial Hospital, Keelung, TW; 6 Department of Cardiology, Dupuytren University Hospital, Limoges, FR; 7 INSERM 1094, Tropical Neuroepidemiology, Limoges University, Limoges, FR; 8 Division of Cardiology and Department of Internal Medicine, Kaohsiung Chang Gung Memorial Hospital, Chang Gung University College of Medicine, Kaohsiung, TW

**Keywords:** hyperthyroidism, atrial fibrillation, thromboembolism, ischemic stroke

## Abstract

**Background:**

Patients with hyperthyroidism have higher risk of atrial fibrillation (AF). However, the risk of thromboembolic event in patients with hyperthyroidism-related AF is controversial.

**Objectives:**

The aim of the study was to examine the risk of thromboembolic events in AF patients with/without hyperthyroidism.

**Methods:**

The national retrospective cohort study enrolled AF population was derived from the Taiwan National Health Insurance Research Database. The comparison between the AF patients with clinical hyperthyroidism (HT-AF group) and AF patients without hyperthyroidism (non-thyroid AF group) was made in a propensity score matched cohort and in a real-world setting, of which, the CHA_2_DS_2_-VASc level was treated as a stratum variable. The outcomes were ischemic stroke and systemic thromboembolism.

**Results:**

There were 3,880 patients in HT AF group and 178,711 in non-thyroid AF group. After propensity score analysis, the incidence of thromboembolism event and ischemic stroke were lower in HT AF patients than non-thyroid AF patients (1.6 versus 2.2 events per 100 person-years; HR, 0.73; 95% CI, 0.64–0.82 and 1.4 versus 1.8 events per 100 person-years; HR, 0.74; 95% CI, 0.64–0.84, respectively) in the 4.3 ± 3.2 year follow up period. The differences persistently existed in those receiving anticoagulants or not. In AF patients without anticoagulants, the incidence densities of ischemic stroke/systemic thromboembolism were significantly lower in HT AF group than those in non-thyroid AF group at CHA_2_DS_2_-VASc scores ≤ 4 (HR, 0.41; 95% CI, 0.35–0.48, p < 0.001), while the differences disappeared in case of score ≥ 5 (HR, 0.80; 95% CI, 0.63–1.02, p = 0.071).

**Conclusion:**

Patients with HT AF had lower incidence of thromboembolic events as compared to non-thyroid AF patients. The threshold of CHA_2_DS_2_-VASc score for anticoagulation in AF patients with clinical hyperthyroidism should be further evaluated.

**Highlights:**

- The incidence of thromboembolic event was different between hyperthyroidism-related atrial fibrillation (HT-AF) and non-thyroid AF patients.
- Hyperthyroidism did not confer additional risk of thromboembolic event at CHA_2_DS_2_-VASc of ≤ 4.
- The benefit of anticoagulation strategy in patients with hyperthyroidism-related AF should be further evaluated, especially at low CHA_2_DS_2_-VASc score.

## Introduction

Atrial fibrillation (AF) is a complication of hyperthyroidism [1,2], and up to 13% of patients with new-onset AF have biochemical evidence of hyperthyroidism [3]. AF occurs in 5% to 15% of patients with hyperthyroidism compared to 1.5% to 2% in the general population [2,4]. However, individuals with hyperthyroidism-related AF differ from those with non-thyroid AF, with higher proportions of women and younger age in the former. In terms of stroke, hyperthyroidism induces hypercoagulable state, endothelial dysfunction, and high inflammation status which contributes to the incidence of thromboembolic events [5,6,7]. In addition, AF is also a well-known risk factor for thromboembolism, and is important to prevent thromboembolism in the management of AF [8]. A few studies have reported that individuals with hyperthyroid AF have a higher risk of stroke than those with non-thyroid AF [5,9,10]. However, there are currently no recommendations focusing on AF patients with clinical hyperthyroidism (HT AF) [8]. In addition, AF related to hyperthyroidism is sometimes considered to be reversible atrial tachyarrhythmia due to the high maintenance rate of sinus rhythm after controlling thyroid hormone [11], and because hypercoagulable and endothelial function can also be improved after correcting hyperthyroidism [6]. Moreover, better adherence to antithyroid drugs was reported to result in a lower risk of stroke in patients with hyperthyroidism in a national cohort study [12].

The CHA_2_DS_2_-VASc score is used to predict the risk of stroke/systemic embolism in non-valvular AF patients: Anticoagulation therapy is recommended in patients at CHA_2_DS_2_-VASc score ≥ 2 (this treatment can also be considered in case of a CHADS2-VASc of 1) [8]. This study hypothesized that the risk of thromboembolism in patients with new-onset HT AF would be lower than that in non-thyroid AF patients. The Taiwan National Health Insurance Research Database (NHIRD) was utilized–a large national population-based database of medical records–to compare differences in the risk of thromboembolism between HT AF patients and non-thyroid AF patients [13].

## Methods

### Study cohort

**Figure 1:**
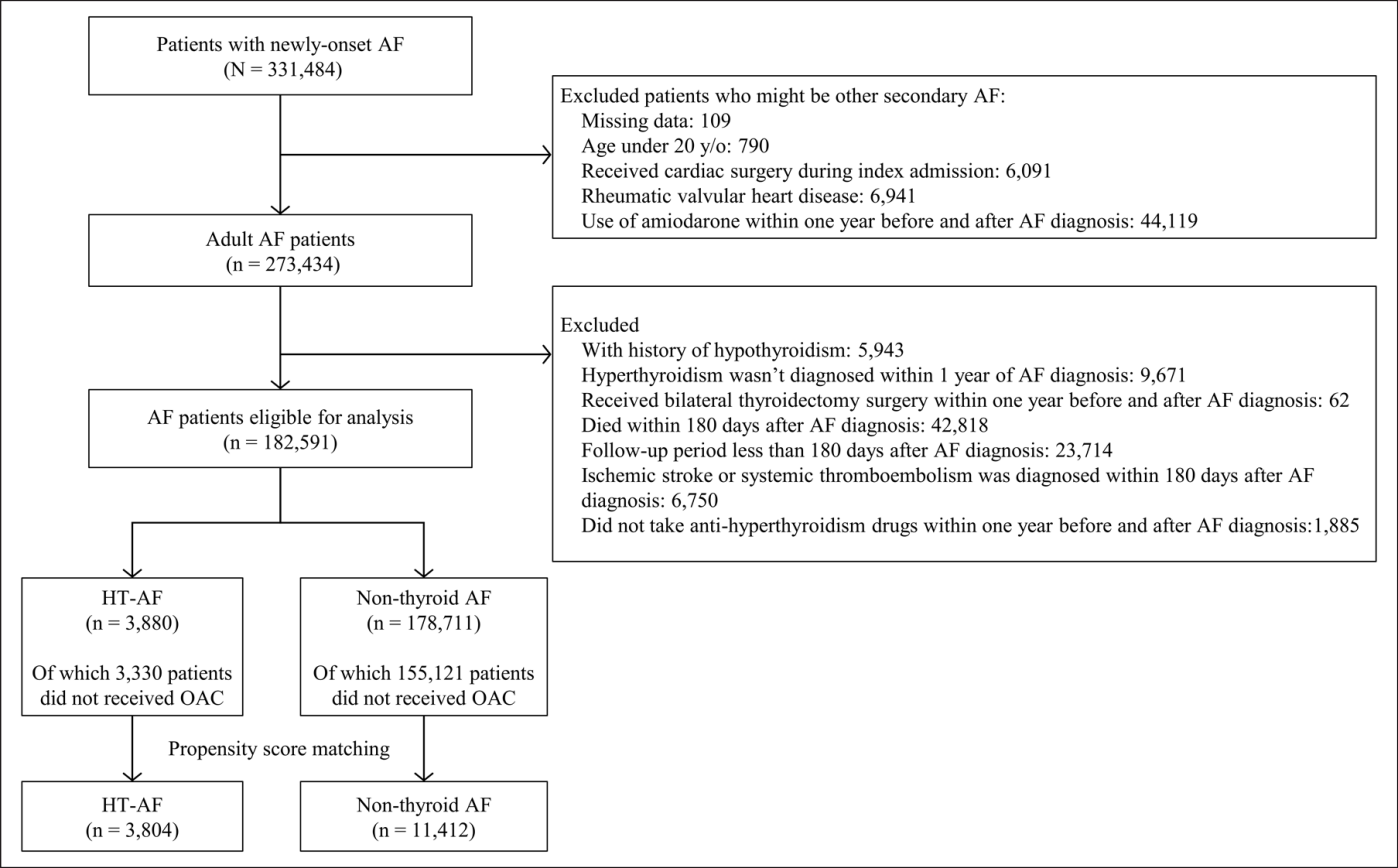
Flowchart of the study.

This was a retrospective analysis derived from the NHIRD. A total of 331,484 patients with new-onset AF (International Classification of Diseases, Ninth Revision, Clinical Modification (ICD-9-CM) code 427.31) were identified from the NHIRD between January 1, 2001 and December 31, 2013. We excluded patients with possible other secondary AF and valvular AF, including those aged <20 years, those whose index diagnosis of AF was during hospitalization for cardiac surgery, and those who were diagnosed with rheumatic valvular heart disease. Those under amiodarone within 1 year before and after a diagnosis of AF were excluded due to the potential effects of this drug on thyroid function. In order to exclude patients with subclinical hyperthyroidism and those with a missing diagnosis of hyperthyroidism, those with a diagnosis of hyperthyroidism but without any prescriptions for anti-hyperthyroidism therapy within 1 year before the index date and the date of diagnosis of hyperthyroidism beyond 1 year before the diagnosis of AF were not accepted. Those with any history of hypothyroidism and those who had received total thyroidectomy within 1 year before and after a diagnosis of AF were excluded. To guarantee receiving adequate anti-hyperthyroidism therapy, those who died or had a thromboembolic event of interest within 180 days after the index date were excluded. The eligible patients were classified into two groups as the HT AF group and those with non-thyroid AF (non-thyroid AF group) (Figure 1). This study was approved by the Institutional Review Board of Chang Gung Memorial Hospital (201700281B1).

### Comorbidities and outcome definition

Baseline comorbidities including hypertension, diabetes mellitus, chronic kidney disease, dyslipidemia, obstructive lung disease, ischemic heart disease, peripheral artery disease, and stroke were recorded with ICD-9-CM diagnosis codes during the index hospitalization and at least two consecutive clinic visits in the previous year before the index date. The index date was defined as the date when AF was first diagnosed and the observation period ended at the time of death, time of clinical outcome events, or on December 31, 2013. The primary outcome of interest was the occurrence of a thromboembolic event including ischemic stroke and systemic thromboembolism that was defined as the principal diagnosis for the hospitalization. Systemic thromboembolism was defined as the vascular thromboembolic occlusion of an extremity or extracranial vital organ.

### Validation of atrial fibrillation, ischemic stroke and systemic thromboembolism in NHIRD

The validation of AF in the NHIRD has been assessed previously, with a positive predictive value (PPV) of 89% [14]. In addition, a previous study with 1,736 consecutive acute ischemic stroke patients validated acute ischemic stroke with a PPV of 88.4% and sensitivity of 97.3% [15]. Furthermore, a validation study for systemic thromboembolism was conducted at the medical center used by this team, and 120 hospitalizations for systemic thromboembolism in AF patients were randomly selected using the same criteria as in this study. After experienced physicians (YSL and VCCW) reviewed the medical records and all imaging results–including vascular duplex, computed tomography angiography, and intervention reports–the PPV of systemic thromboembolism was 86.7%.

### Statistics

The baseline characteristics of the HT AF and non-thyroid AF groups were compared using the independent samples t-test for continuous variables and the chi-square test for categorical variables. The risks of mortality and thromboembolic events between the two groups were compared using a Cox proportional hazard model. The Cox models were first conducted in a propensity score-matched cohort, with each patient in the HT AF group being matched to three counterparts in the non-thyroid AF group according to the propensity score calculated based on age, sex, CHA_2_DS_2_-VASc components, and non-antithyroid drugs. The matching was processed using a greedy nearest neighbor algorithm with a caliper of 0.2 times of the standard deviation of the logit of propensity score, with random matching order and without replacement. Furthermore, we matched the HT AF patients with non-thyroid AF patients in the cohort with or without oral anticoagulation therapy separately. Cox models were also used in a real-world setting in which the CHA_2_DS_2_-VASc score was treated as a stratum variable when comparing the risks of thromboembolic events between the HT AF and non-thyroid AF groups. The study group (HT AF versus non-thyroid AF) was the only explanatory variable in the Cox models. The level of statistical significance was set as 0.05 and no adjustment of multiple testing (multiplicity) was made to avoid low statistical power in this study. Data analysis was conducted using SAS software version 9.4 (SAS Institute, Cary, NC), including the procedures of ‘psmatch’ for propensity score matching and ‘phreg’ for survival analysis.

## Results

### Baseline characteristics

From January 1, 2001 to December 31, 2013, a total of 331,484 patients with newly diagnosed AF were identified. After applying the exclusion criteria, a total of 182,591 patients were included, of whom 3,880 had HT AF and 178,711 had non-thyroid AF (Figure 1). The baseline characteristics are listed in Table 1. There were higher prevalence rates of nearly all comorbidities in the non-thyroid AF group compared to the HT AF group. In addition, the patients in the HT AF group were younger (Table 1). Similarly, the non-thyroid AF group used more medications, except for beta-blockers.

**Table 1:** Real-world baseline characteristics and medications of the patients with atrial fibrillation with and without clinical hyperthyroidism before matching.

Variables	The whole AF population			AF population without OAC		

	HT AF (*n* = 3,880)	Non-HT AF (*n* = 178,711)	*P*-value	HT AF (*n* = 3,330)	Non-HT AF (*n* = 155,121)	*P*-value

Age (years, mean, SD)	60.1 ± 14.8	71.9 ± 13.6	<0.001	59.9 ± 14.9	72.1 ± 13.7	<0.001
Sex			<0.001			<0.001
Male	1,355 (34.9)	101,435 (56.8)		1,139 (34.2)	87,791 (56.6)	
Comorbidities						
Dyslipidemia	202 (5.2)	18,422 (10.3)	<0.001	165 (5.0)	15,495 (10.0)	<0.001
Chronic kidney disease	232 (6.0)	22,502 (12.6)	<0.001	198 (5.9)	20,145 (13.0)	<0.001
Obstructive pulmonary disease	379 (9.8)	35,923 (20.1)	<0.001	322 (9.7)	32,618 (21.0)	<0.001
Ischemic heart disease	886 (22.8)	63,793 (35.7)	<0.001	753 (22.6)	55,482 (35.8)	<0.001
Peripheral arterial disease	57 (1.5)	4,712 (2.6)	<0.001	47 (1.4)	4,014 (2.6)	<0.001
History of stroke	223 (5.7)	26,797 (15.0)	<0.001	185 (5.6)	23,080 (14.9)	<0.001
History of myocardial infarction	57 (1.5)	6,778 (3.8)	<0.001	48 (1.4)	5,929 (3.8)	<0.001
Malignancy	135 (3.5)	10,549 (5.9)	<0.001	120 (3.6)	9,373 (6.0)	<0.001
Components of CHA_2_DS _2_-VASc						
Congestive heart failure	240 (6.2)	21,632 (12.1)	<0.001	198 (5.9)	18,741 (12.1)	<0.001
Hypertension	1,738 (44.8)	100,341 (56.1)	<0.001	1,485 (44.6)	85,996 (55.4)	<0.001
Age (years)			<0.001			<0.001
65–74	765 (19.7)	45,486 (25.5)		645 (19.4)	38,488 (24.8)	
≥75	724 (18.7)	86,584 (48.4)		610 (18.3)	76,945 (49.6)	
Diabetes mellitus	548 (14.1)	31,986 (17.9)	<0.001	466 (14.0)	27,738 (17.9)	<0.001
Prior stroke or systemic thromboembolism	252 (6.5)	29,643 (16.6)	<0.001	204 (6.1)	25,053 (16.2)	<0.001
Vascular disease	927 (23.9)	67,315 (37.7)	<0.001	787 (23.6)	58,471 (37.7)	<0.001
Sex: female	2,525 (65.1)	77,276 (43.2)	<0.001	2,191 (65.8)	67,330 (43.4)	<0.001
CHA_2_DS_2_-VASc score			<0.001			<0.001
0	532 (13.7)	12,857 (7.2)		465 (14.0)	11,283 (7.3)	
1	1,134 (29.2)	21,302 (11.9)		986 (29.6)	18,249 (11.8)	
2	751 (19.4)	30,426 (17.0)		644 (19.3)	26,297 (17.0)	
3	552 (14.2)	37,148 (20.8)		464 (13.9)	32,311 (20.8)	
4	445 (11.5)	33,687 (18.8)		377 (11.3)	29,383 (18.9)	
5	276 (7.1)	22,620 (12.7)		226 (6.8)	19,696 (12.7)	
6	114 (2.9)	12,359 (6.9)		104 (3.1)	10,690 (6.9)	
7–9	76 (2.0)	8,312 (4.6)		64 (2.0)	7,212 (4.6)	
Total score	2.2 ± 1.8	3.2 ± 1.9	<0.001	2.2 ± 1.8	3.2 ± 1.9	<0.001
Medication						
ACEI/ARB	1,491 (38.4)	75,769 (42.4)	<0.001	1,210 (36.3)	62,690 (40.4)	<0.001
CCB	682 (17.6)	48,664 (27.2)	<0.001	605 (18.2)	42,425 (27.3)	<0.001
β-blocker	2,809 (72.4)	61,562 (34.4)	<0.001	2,408 (72.3)	51,644 (33.3)	<0.001
Statin	197 (5.1)	19,519 (10.9)	<0.001	154 (4.6)	15,674 (10.1)	<0.001
Biguanides	382 (9.8)	18,936 (10.6)	0.133	318 (9.5)	16,090 (10.4)	0.123
Sulfonylurea	392 (10.1)	20,899 (11.7)	0.002	339 (10.2)	18,042 (11.6)	0.010
DPP4i	76 (2.0)	2,983 (1.7)	0.164	60 (1.8)	2,407 (1.6)	0.249
Insulin	106 (2.7)	6,125 (3.4)	0.018	93 (2.8)	5,384 (3.5)	0.034
Anticoagulants	550 (14.2)	23,590 (13.2)	0.076	NA	NA	NA
Anti-hyperthyroidism agents						
Methimazole	1,556 (40.1)	0 (0.0)	NA	922 (27.7)	0 (0.0)	NA
Carbimazole	455 (11.7)	0 (0.0)	NA	681 (20.5)	0 (0.0)	NA
Propylthiouracil	814 (21.0)	0 (0.0)	NA	374 (11.2)	0 (0.0)	NA
Mixed (switch)	1,055 (27.2)	0 (0.0)	NA	1,353 (40.6)	0 (0.0)	NA

ACEI, angiotensin-converting enzyme inhibitors; AF, atrial fibrillation; ARB, angiotensin receptor blockers; CCB, calcium channel blockers; DPP4i, dipeptidyl peptidase 4 inhibitor; HT, hyperthyroidism; OAC, oral anticoagulants; NA, not applicable; SD, standard.

### Thromboembolic events between the HT AF and non-thyroid AF groups

**Figure 2:**
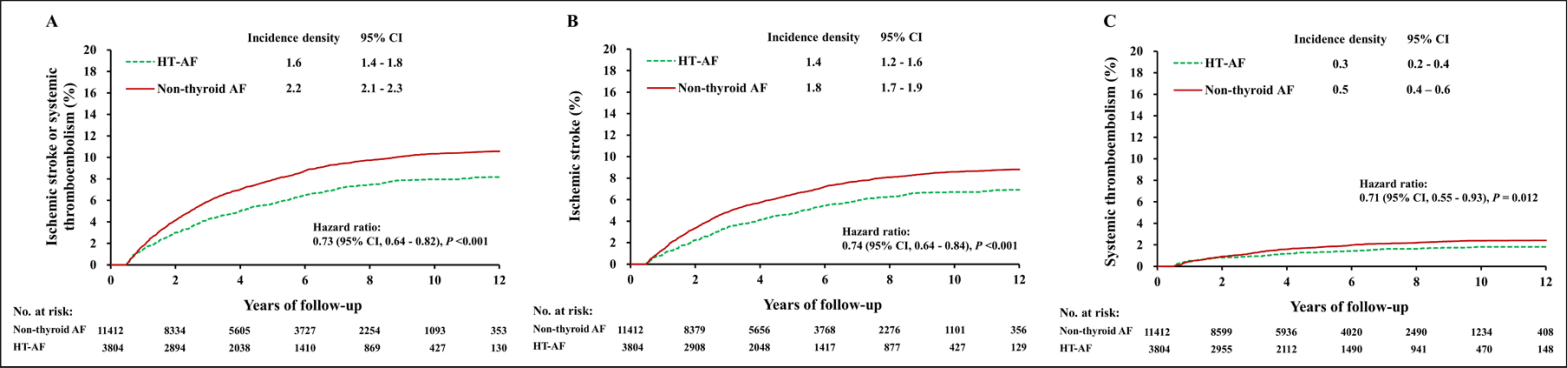
Cumulative event rates of ischemic stroke or systemic thromboembolism **(A)**, ischemic stroke **(B)**, and systemic thromboembolism **(C)** in AF patients with clinical hyperthyroidism and non-thyroid AF patients in a propensity score-matched cohort. AF: atrial fibrillation.

Systemic thromboembolism and ischemic stroke were evaluated over a follow-up period of 4.3 ± 3.2 years and analyzed after propensity matching in order to adjust for disparities in baseline characteristics between groups. There were 3,804 patients in the HT AF group and 11,412 patients in the non-thyroid AF group (Supplemental Table S1). In terms of thromboembolic events, the incidence was lower in the HT AF group than in the non-thyroid AF group (1.6 versus 2.2 events per 100 person-years; hazard ratio [HR], 0.73; 95% confidence interval [CI], 0.64–0.82) (Figure 2A). When the incidence of thromboembolic events were assessed separately, the incidence of ischemic stroke was lower in the HT AF group than in the non-thyroid AF group (1.4 versus 1.8 events per 100 person-years; HR, 0.74; 95% CI, 0.64–0.84) (Figure 2B), and the trend was the same in the comparison of systemic thromboembolism (0.3 versus 0.5 events per 100 person-years; HR, 0.71; 95% CI, 0.55–0.93) (Figure 2C).

Regarding the mortality, the incidence of mortality was lower in the HT AF patients than that in the non-thyroid AF patients. (3.7 versus 10.2 events per 100 person-years; HR, 0.36; 95% confidence interval [CI], 0.34–0.39) and similar results can be observed after propensity score matching (3.7 versus 5.0 events per 100 person-years; HR, 0.74; 95% CI, 0.68–0.80) (Supplemental Figure S1).

### Thromboembolic events in subgroups with/without anticoagulation therapy

**Figure 3:**
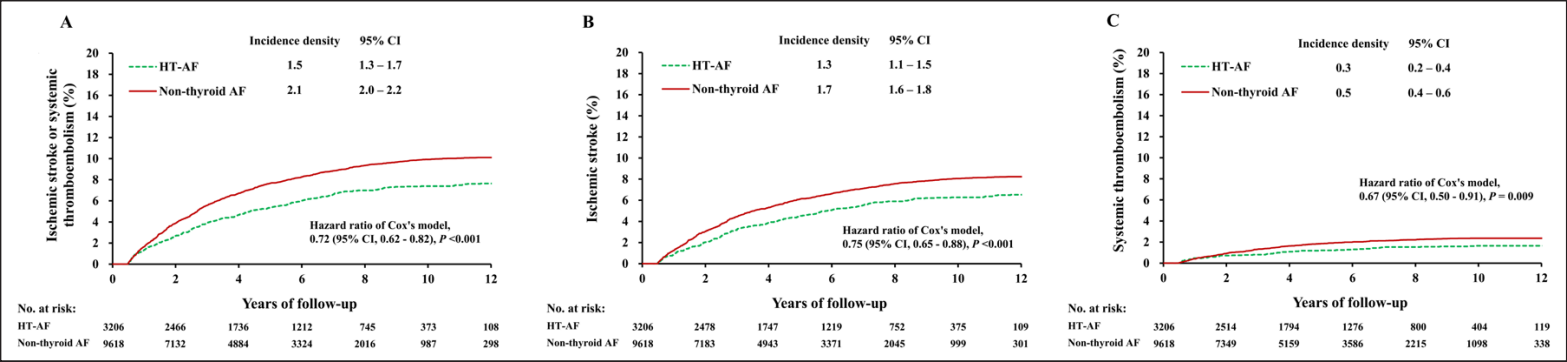
Cumulative event rates of ischemic stroke or systemic thromboembolism **(A)**, ischemic stroke **(B)**, and systemic thromboembolism **(C)** in AF patients with clinical hyperthyroidism and non-thyroid AF patients who were not prescribed with anticoagulants after propensity score matching. AF: atrial fibrillation.

**Figure 4:**
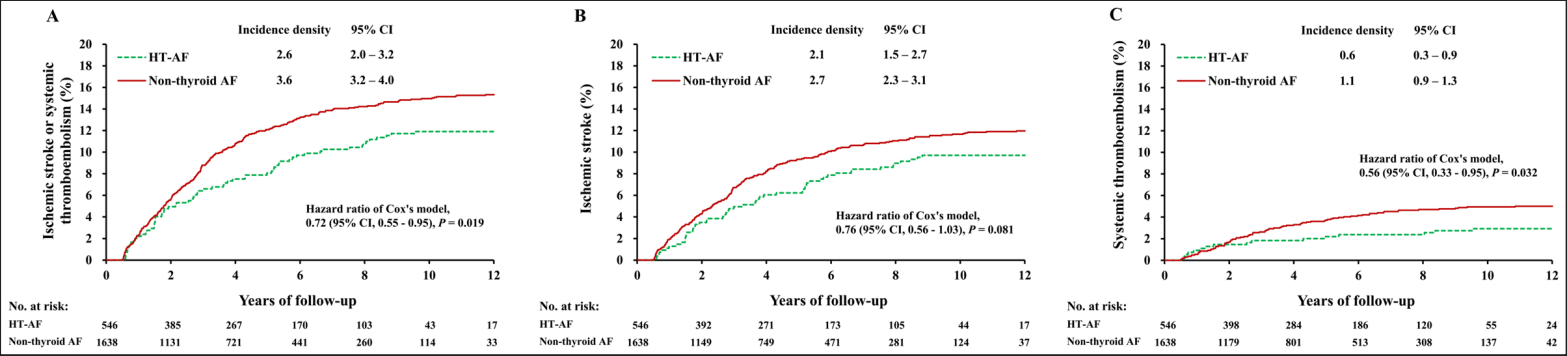
Cumulative event rates of ischemic stroke or systemic thromboembolism **(A)**, ischemic stroke **(B)**, and systemic thromboembolism **(C)** in AF patients with clinical hyperthyroidism and non-hyperthyroidism AF patients who were prescribed with anticoagulants after propensity score matching. AF: atrial fibrillation.

The thromboembolic events in the patients with and without prescriptions for anticoagulants were analyzed after propensity matching and the baseline characteristics are listed in Supplemental Table S1. In the patients without anticoagulation therapy, the incidence of thromboembolic event was still significantly lower in the HT AF group than in the non-thyroid AF group (1.5 versus 2.1 events per 100 person-years; HR, 0.72; 95% CI, 0.62–0.82) (Figure 3A). The trend was similar in the comparisons of ischemic stroke and systemic thromboembolic events (Figure 3B, 3C). In the patients with anticoagulation therapy, the incidence of thromboembolic event was also significantly lower in the HT AF group than in the non-thyroid AF group (2.6 versus 3.6 events per 100 person-years; HR, 0.72; 95% CI, 0.55–0.95) (Figure 4A). The difference existed in terms of systemic thromboembolism and it has a trend in terms of ischemic stroke (Figure 4B, 4C).

In addition, the incidence of thromboembolic events was analyzed in HT patients receiving different antithyroid drugs (including methimazole, carbimazole, and propylthiouracil) by excluding those who used two or more antithyroid drugs during the observation period. The risks of ischemic stroke among those receiving methimazole, carbimazole, or propylthiouracil were not significantly different, neither in the whole cohort nor in those stratified according to the use of anticoagulants (Supplemental Figure S2).

### Thromboembolic events according to CHA2DS2-VASc score in patients without anticoagulant therapy

**Figure 5:**
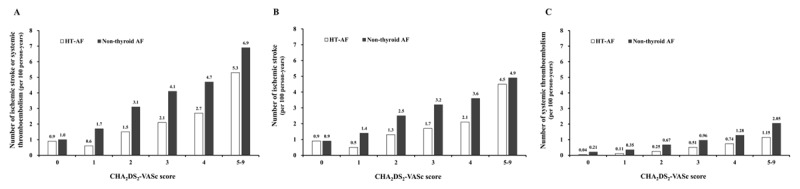
Incidence densities of ischemic stroke or systemic thromboembolism **(A)**, ischemic stroke **(B)**, and systemic thromboembolism **(C)** in AF patients with clinical hyperthyroidism and non-thyroid AF patients in a real-world setting according to CHA_2_DS_2_-VASc score. AF: atrial fibrillation.

As shown in Table 1, the distribution of CHA_2_DS_2_-VASc scores differed in the two groups. The incidence density (ID) of thromboembolic event (Figure 5A) and ischemic stroke (Figure 5B) were significantly lower in the HT AF group than in the non-thyroid AF group with CHA_2_DS_2_-VASc scores of 1–4, but there was no significant difference in those with a CHA_2_DS_2_-VASc score ≥ 5 (Table 2). However, the incidence of systemic thromboembolism in the non-thyroid AF group was significantly higher than that in the HT AF group in both those with a CHA_2_DS_2_-VASc score ≤ 4 and ≥ 5 (Figure 5C) (Table 2). Importantly, those with HT-AF with a CHA_2_DS_2_-VASc score of 1 had very low rates of thromboembolic events, ischemic stroke, and systemic thromboembolism (ID: 0.6 events, 0.5 and 0.11 per 100 person-years, respectively), compared to the non-thyroid AF patients with a score of 0 (ID: 1.7 events, 1.4 and 0.35 per 100 person-years, respectively) (Figures 5A–5C). In terms of mortality, the incidence of mortality was higher in non-thyroid AF group than that in HT AF group in any level of CHA_2_DS_2_-VASc. (Supplemental Table S2)

**Table 2:** Detailed information of Ischemic Stroke/Systemic Thromboembolism, Ischemic Stroke and Systemic Thromboembolism in AF patients with and without clinical hyperthyroidism who did not prescribe any anticoagulation therapy.

Ischemic stroke/ Systemic thromboembolism								

CHA_2_DS_2_-VASc	HT AF			Non-HT AF			HT AF *vs*. Non-HT AF	

	Numbers	Event (%)	ID (95% CI) §	Numbers	Event (%)	ID (95% CI) §	HR (95% CI)	*P*-value

0	465	21 (4.5)	0.9 (0.5–1.3)	11283	583 (5.2)	1.0 (0.9–1.1)	0.82 (0.53–1.27)	0.376
1	986	32 (3.3)	0.6 (0.4–0.8)	18249	1462 (8.0)	1.7 (1.6–1.8)	0.36 (0.25–0.51)	<0.001
2	644	45 (7.0)	1.5 (1.1–1.9)	26297	3083 (11.7)	3.1 (3.0–3.2)	0.49 (0.36–0.65)	<0.001
3	464	43 (9.3)	2.1 (1.5–2.7)	32311	4512 (14.0)	4.1 (4.0–4.2)	0.52 (0.39–0.71)	<0.001
4	377	39 (10.3)	2.7 (1.8–3.6)	29383	4311 (14.7)	4.7 (4.6–4.8)	0.58 (0.42–0.80)	<0.001
5–9	394	67 (17.0)	5.3 (4.0–6.6)	37598	6800 (18.1)	6.9 (6.7–7.1)	0.80 (0.63–1.02)	0.071
**Stratified**								
0–4	2936	180 (6.1)	1.3 (1.1–1.5)	117523	13951 (11.9)	3.1 (3.0–3.2)	0.41 (0.35–0.48)	<0.001
5–9	394	67 (17.0)	5.3 (4.0–6.6)	37598	6430 (17.1)	6.9 (6.7–7.1)	0.80 (0.63–1.02)	0.071
**Total**	**3330**	**247 (7.4)**	**1.6 (1.4–1.8)**	**155121**	**20381 (13.1)**	**3.9 (3.8–4.0)**	**0.44 (0.38–0.49)**	**<0.001**
**Ischemic Stroke**								

0	465	21 (4.5)	0.9 (0.5–1.3)	11283	487 (4.3)	0.9 (0.8–1.0)	0.99 (0.64–1.53)	0.954
1	986	28 (2.8)	0.5 (0.3–0.7)	18249	1226 (6.7)	1.4 (1.3–1.5)	0.37 (0.26–0.54)	<0.001
2	644	39 (6.1)	1.3 (0.9–1.7)	26297	2551 (9.7)	2.5 (2.4–2.6)	0.51 (0.37–0.70)	<0.001
3	464	35 (7.5)	1.7 (1.1–2.3)	32311	3627 (11.2)	3.2 (3.1–3.3)	0.53 (0.38–0.74)	<0.001
4	377	31 (8.2)	2.1 (1.3–2.9)	29383	3316 (11.3)	3.6 (3.5–3.7)	0.60 (0.42–0.86)	0.005
5–9	394	57 (14.5)	4.5 (3.3–5.7)	37598	4747 (12.6)	4.9 (4.8–5.0)	0.93 (0.72–1.21)	0.600
**Stratified**								
0–4	2936	154 (5.3)	1.1 (0.9–1.3)	117523	11207 (9.5)	2.5 (2.5–2.5)	0.44 (0.38–0.52)	<0.001
5–9	394	57 (14.5)	4.5 (3.3–5.7)	37598	4747 (12.6)	4.9 (4.8–5.0)	0.93 (0.72–1.21)	0.600
**Total**	**3330**	**211 (6.3)**	**1.4 (1.2–1.6)**	**155121**	**15954 (10.3)**	**2.9 (2.9–2.9)**	**0.48 (0.42–0.55)**	**<0.001**
**Systemic Thromboembolism**								

0	465	1 (0.2)	0.04 (–0.04–0.12)	11283	123 (1.1)	0.21 (0.17–0.25)	0.19 (0.03–1.33)	0.093
1	986	6 (0.6)	0.11 (0.02–0.20)	18249	313 (1.7)	0.35 (0.31–0.39)	0.32 (0.14–0.72)	0.006
2	644	8 (1.2)	0.25 (0.07–0.43)	26297	721 (2.7)	0.67 (0.62–0.72)	0.38 (0.19–0.76)	0.006
3	464	11 (2.4)	0.51 (0.21–0.81)	32311	1139 (3.5)	0.96 (0.90–1.02)	0.54 (0.30–0.98)	0.042
4	377	11 (2.9)	0.74 (0.30–1.18)	29383	1254 (4.3)	1.28 (1.21–1.35)	0.58 (0.32–1.05)	0.074
5–9	394	16 (4.1)	1.15 (0.59–1.71)	37598	2123 (5.7)	2.05 (1.96–2.14)	0.58 (0.36–0.95)	0.030
**Stratified**								
0–4	2936	37 (1.3)	0.25 (0.17–0.33)	117523	3550 (3.0)	0.75 (0.73–0.77)	0.34 (0.25–0.47)	<0.001
5–9	394	16 (4.1)	1.15 (0.59–1.71)	37598	2123 (5.7)	2.05 (1.96–2.14)	0.58 (0.36–0.95)	0.030
**Total**	**3330**	**53 (1.6)**	**0.33 (0.24–0.42)**	**155121**	**5673 (3.7)**	**0.99 (0.96–1.02)**	**0.35 (0.26–0.45)**	**<0.001**

AF, atrial fibrillation; HT, hyperthyroidism; ID, incidence density; CI, confidence interval; HR, hazard ratio. § Incidence density: Number of events per 100 person-years.

## Discussion

This is the first large cohort study to evaluate the incidence of thromboembolism in patients with AF according to thyroid status using propensity score-matched analysis to compensate for baseline disparities. Results showed that the incidence rates of ischemic stroke and systemic thromboembolism were lower in the patients with HT AF than in those with non-thyroid AF, whether or not they were receiving anticoagulants. In addition, antithyroid medication did not alter the incidence of thromboembolism. In terms of ischemic stroke and thromboembolic event risk stratification, according to CHA_2_DS_2_-VASc score, the incidence rates in the HT AF group were significantly lower in those with a CHA_2_DS_2_-VASc score of ≤ 4 but comparable to those with a CHA_2_DS_2_-VASc score ≥ 5 in the non-thyroid AF group.

Atrial fibrillation in patients with clinical hyperthyroidism is often considered as reversible, with a high sinus rhythm maintenance rate after the restoration of thyroid function compared to those with non-hyperthyroid AF [11,16,17]. Thyroid hormone increases automaticity and enhances triggered activity of the cardiomyocytes of pulmonary veins, and this may increase the arrhythmogenic activity of pulmonary veins and thus induce the occurrence of AF [18]. This hinted at a lower burden of AF in patients with hyperthyroidism when the thyroid is in a euthyroid status [11]. In terms of burden of AF, it was reported to have high correlation with thromboembolic event from patients with cardiac implanted electronic devices implantation [19]. On the other hand, it was documented that long-term risk of ischemic stroke was lower in patients with AF occurring with stress than those with a history of AF from study enrolled patients admitted for sepsis [20]. This explained why the long-term risk of thromboembolism was significantly lower in HT AF group than non-thyroid AF group in this study though some studies showed higher thromboembolic event at the initial phase in hyperthyroidism patients with thyrotoxic AF [10].

One small prospective single center study reported a higher incidence of ischemic stroke in hyperthyroidism patients who presented with new-onset AF than in those without AF after 1 year of follow-up, after matching for age and gender [10]. However, another observational study that included AF patients did not find that hyperthyroidism was an independent risk factor for stroke or systemic thromboembolism in a 10-year follow-up period [21]. Moreover, another observational AF cohort study enrolled 642 hyperthyroid AF patients, and found that the patients with hyperthyroidism AF had a lower risk of stroke compared to their non-thyroid counterparts with the same CHA_2_DS_2_-VASc score [22]. Taken together, these studies suggest that hyperthyroidism may be a risk factor contributing to thromboembolism, but that AF plays a key role in thromboembolism, including ischemic stroke, in long-term follow up. However, these studies had some limitations, including a small sample size favoring heterogeneous conclusions and no adjustments for several comorbidities, even different baseline comorbidities between them. In addition, hyperthyroidism patients who present with AF should be treated, but this was not considered in these studies. To fill these gaps and to reduce confounding bias as far as possible, this study enrolled a large nationwide cohort. Based on reimbursements of the Taiwan National Health Insurance program, antithyroid therapy should be prescribed based on the clinical presentation and disease relevance. Therefore, around 5~6% of the AF patients had concomitant hyperthyroidism–or had a history of–and around 1% of those with new-onset AF also had a clinical diagnosis of hyperthyroidism in our study. This prevalence is similar to previous reports, in which 5~15% of patients with AF had hyperthyroidism, but fewer than 1% had new-onset AF caused by hyperthyroidism [2]. Of note, the presence of hyperthyroidism did not confer an additional risk of thromboembolic events compared with non-thyroid AF in the patients with a CHA_2_DS_2_-VASc score ≤ 4 in this study which was compatible to those with a CHA_2_DS_2_-VASc score ≥ 5. In addition, while the major drugs used to treat hyperthyroidism, including methimazole, carbimazole, and propylthiouracil are metabolized through the liver, none of the previous reports mentioned the impact of these drugs on thromboembolism, except for hepatoxicity [23,24]. As anticoagulants are mainly metabolized in the liver, a subgroup analysis was performed of those receiving different antithyroid therapies in those with/without anticoagulation therapy which found that the incidence of thromboembolic events may be not influenced by antithyroid therapies.

Furthermore, more than 80% of the AF patients did not receive anticoagulation therapy in the enrolled population, which allowed assessment of the “natural” incidence of thromboembolism. According to previous reports on the prevalence of the underuse of anticoagulation therapy in Asia, including Taiwan, only around 20~25% of AF patients receive anticoagulation therapy [25,26]. In terms of anticoagulation therapy in patients with hyperthyroidism-related AF in Asia, the prevalence varies from 6% in China [5] to around 20% in Hong Kong [22]. Further studies should be conducted in HT AF patients with anticoagulants.

The results of this study have two clinical implications: First, it provides strong evidence that the incidence of thromboembolic events differs between hyperthyroidism-related AF patients and non-thyroid AF patients, regardless of whether or not they receive anticoagulants. In addition, it was found that different antithyroid drugs did not influence the incidence of thromboembolic events in HT AF patients. Second, current guidelines mandate the use of anticoagulants in patients with a CHA_2_DS_2_-VASc score ≥ 2 and also suggest their use in patients with a CHA_2_DS_2_-VASc score of 1, but do not mention whether thyroid function should alter the strategy of preventing thromboembolic events in AF patients However, the incidence of thromboembolic events, including ischemic stroke, was lower than 1% in the HT AF patients with a CHA_2_DS_2_-VASc score £ 1 in this study. Several clinical trials have reported that direct oral anticoagulants should be considered when the annual incidence of stroke is ≥ 0.9% [27]. Therefore, the strategy of anticoagulation therapy for AF patients with clinical hyperthyroidism should be reconsidered and investigated further, especially in those with a CHA_2_DS_2_-VASc score of 1.

There are several limitations to this study. First, it could not be clearly confirmed that hyperthyroidism was the direct cause of AF as it was not possible to access the primary medical records of all patients. This study tried to increase the likelihood that AF in our patients was related to hyperthyroidism by enrolling patients with new-onset AF and a diagnosis of hyperthyroidism around the same time as the diagnosis of AF. This study also excluded patients under amiodarone therapy within 1 year before enrollment. Second, this study was unable to differentiate different types of AF because this information is not available in the NHIRD. However, the incidence of ischemic stroke is considered to be lower in paroxysmal AF patients than in those with sustained AF [28], and the different types of AF should not change the anticoagulation strategy [8]. In addition, Wong et al. reported that hyperthyroid AF patients who had a restored sinus rhythm had a lower incidence of stroke than those with persistent AF [29]. In this study, information on sinus rhythm restoration was lacking. Third, as laboratory data are not available in the NHIRD, thyroid function could not be assessed, and so subclinical hyperthyroidism or the severity of hyperthyroidism could not be clearly classified. Although the level of thyroxine may affect the restoration of sinus rhythm [30] and influence endothelial function and coagulation status [6], two strategies were used to minimize these limitations. The first strategy was to include antithyroid drugs as one of the selection criteria in the HT AF group to increase the likelihood of a correct diagnosis of hyperthyroidism, and the second was to count the number of clinical events after 6 months of antithyroid therapy under the assumption that a euthyroid status had been achieved.

## Conclusion

In this study, the patients with hyperthyroidism-associated AF had a lower risk of thromboembolic events, including ischemic stroke, than those with non-thyroid AF, especially in those with a CHA_2_DS_2_-VASc score of ≤4. Findings suggested the use of anticoagulants might be reconsidered in patients with HT-related AF, especially at low CHA_2_DS_2_-VASc score. Further large database analyses are required to confirm these findings.

## Additional File

The additional file for this article can be found as follows:

10.5334/gh.871.s1 Supplemental file. Tables S1 & S2 and Figures S1 & S2.
