# Supplementary material for: Risk of Thromboembolism in Non-Valvular Atrial Fibrillation With or Without Clinical Hyperthyroidism
Source: Glob Heart. 2021 Jun 17;16(1):45. doi: 10.5334/gh.871 (PMC8212838; doi:10.5334/gh.871)

Table S1. Baseline characteristics of Atrial fibrillation patients with clinical hyperthyroidism and non-thyroid after matching.

	All			OAC			Non-OAC		
	HT AF	Non-thyroid AF		HT AF	Non-thyroid AF		HT AF	Non-thyroid AF	
Variables	(n = 3,804)	(n = 11,412)	P-value	(*n* = 546)	(*n* = 1,638)	*P*-value	(*n* = 3,206)	(*n* = 9,618)	*P*-value
Age	60.6±14.6	60.6±15.5	0.886	61.8±13.8	61.6±14.2	0.802	60.6±14.6	60.6±15.7	0.948
Female	2,449 (64.4)	7,416 (65.0)	0.499	330 (60.4)	982 (60.0)	0.840	2,068 (64.5)	6,231 (64.8)	0.773
Components of CHA_2DS_2-VASc									
Congestive heart failure	240 (6.3)	725 (6.4)	0.924	42 (7.7)	110 (6.7)	0.437	198 (6.2)	589 (6.1)	0.915
Hypertension	1,737 (45.7)	5,144 (45.1)	0.529	253 (46.3)	733 (44.7)	0.519	1,480 (46.2)	4,397 (45.7)	0.660
Age			0.930			0.923			0.766
< 65	2,315 (60.9)	6,964 (61.0)		312 (57.1)	946 (57.8)		1,951 (60.9)	5,896 (61.3)	
65-74	765 (20.1)	2,263 (19.8)		120 (22.0)	363 (22.2)		645 (20.1)	1,878 (19.5)	
≥ 75	724 (19.0)	2,185 (19.1)		114 (20.9)	329 (20.1)		610 (19.0)	1,844 (19.2)	
Diabetes mellitus	548 (14.4)	1,621 (14.2)	0.758	82 (15.0)	236 (14.4)	0.726	465 (14.5)	1,401 (14.6)	0.931
Prior stroke or systemic thromboembolism	252 (6.6)	747 (6.5)	0.865	48 (8.8)	138 (8.4)	0.791	204 (6.4)	591 (6.1)	0.657
Vascular disease	927 (24.4)	2,811 (24.6)	0.744	140 (25.6)	385 (23.5)	0.312	786 (24.5)	2,349 (24.4)	0.915
CHA_2DS_2-VASc score			0.721			0.737			0.945
0	532 (14.0)	1,537 (13.5)		67 (12.3)	217 (13.2)		464 (14.5)	1,375 (14.3)	
1	1,059 (27.8)	3,190 (28.0)		144 (26.4)	445 (27.2)		869 (27.1)	2,593 (27.0)	
≥ 2	2,213 (58.2)	6,685 (58.6)		335 (61.4)	976 (59.6)		1,873 (58.4)	5,650 (58.7)	

Total score	2.3±1.8	2.3±1.7	0.978	2.4±1.7	2.3±1.7	0.350	2.3±1.8	2.3±1.7	0.780
Medications									
β-blocker	2,733 (71.8)	8,121 (71.2)	0.419	397 (72.7)	1,160 (70.8)	0.397	2,284 (71.2)	6,784 (70.5)	0.446
HTN medications									
ACEI/ARB	1,467 (38.6)	4,363 (38.2)	0.714	279 (51.1)	849 (51.8)	0.767	1,179 (36.8)	3,484 (36.2)	0.574
CCB	681 (17.9)	1,958 (17.2)	0.293	77 (14.1)	215 (13.1)	0.561	604 (18.8)	1,786 (18.6)	0.734
Statin	197 (5.2)	609 (5.3)	0.707	43 (7.9)	135 (8.2)	0.786	154 (4.8)	449 (4.7)	0.754
Warfarin	543 (14.3)	1,683 (14.7)	0.474	546 (100)	1,638 (100)	NA	0 (0.0)	0 (0.0)	NA
Anti-thyroid drugs									
Methimazole	1531 (40.3)	0 (0.0)	NA	201 (36.8)	0 (0.0)	NA	1,317 (41.1)	0 (0.0)	NA
Carbimazole	448 (11.8)	0 (0.0)	NA	81 (14.8)	0 (0.0)	NA	362 (11.3)	0 (0.0)	NA
Propylthiouracil (PTU)	797 (21.0)	0 (0.0)	NA	131 (24.0)	0 (0.0)	NA	657 (20.5)	0 (0.0)	NA
Mixed	1028 (27.0)	0 (0.0)	NA	133 (24.4)	0 (0.0)	NA	870 (27.1)	0 (0.0)	NA

ACEI, angiotensin-converting enzyme inhibitors; AF, atrial fibrillation; HT, hyperthyroidism; ARB, angiotensin receptor blockers; CCB, calcium channel blockers; NA, not applicable; OAC, oral anticoagulants.

Table S2. The incidence of mortality in AF patients with and without clinical hyperthyroidism who did not prescribe any anticoagulants

**Mortality**								
	HT AF			Non-HT AF			HT AF *vs*. Non-HT AF	
CHA_2DS_2-VASc	Numbers	Event (%)	ID (95% CI) §	Numbers	Event (%)	ID (95% CI) §	HR (95% CI)	*P*-value
0	465	36 (7.7)	1.3 (0.9–1.7)	11283	1530 (13.6)	2.4 (2.3–2.5)	0.54 (0.39 - 0.75)	<0.001
1	986	84 (8.5)	1.4 (1.1–1.8)	18249	3842 (21.1)	3.9 (3.8–4.0)	0.37 (0.30 - 0.46)	<0.001
2	644	89 (13.8)	2.5 (2.0–3.1)	26297	10409 (39.6)	8.5 (8.4–8.7)	0.30 (0.24 - 0.36)	<0.001
3	464	133 (28.7)	5.6 (4.6–6.5)	32311	15484 (47.9)	11.3 (11.1–11.5)	0.49 (0.41 - 0.58)	<0.001
4	377	135 (35.8)	7.9 (6.6–9.2)	29383	15157 (51.6)	13.2 (13.0–13.4)	0.59 (0.50 - 0.70)	<0.001
5-9	394	182 (46.2)	11.3 (9.7–12.9)	37598	22688 (60.3)	18.1 (17.9–18.4)	0.61 (0.53 - 0.71)	<0.001
**Stratified**								
0-4	2936	477 (16.3)	2.9 (2.7–3.2)	117523	46422 (39.5)	8.7 (8.6–8.7)	0.34 (0.31 - 0.37)	<0.001
5-9	394	182 (46.2)	11.3 (9.7–12.9)	37598	22688 (60.3)	18.1 (17.9–18.4)	0.61 (0.53 - 0.71)	<0.001
**Total**	**3330**	**659 (19.8)**	**3.7 (3.4–4.0)**	**155121**	**69110 (44.6)**	**10.5 (10.4–10.5)**	**0.35 (0.33 - 0.38)**	**<0.001**

Figure S1. Cumulative event rates of mortality in the AF patients with clinical hyperthyroidism and non-thyroid AF patients in a propensity score-matched cohort. AF: atrial fibrillation

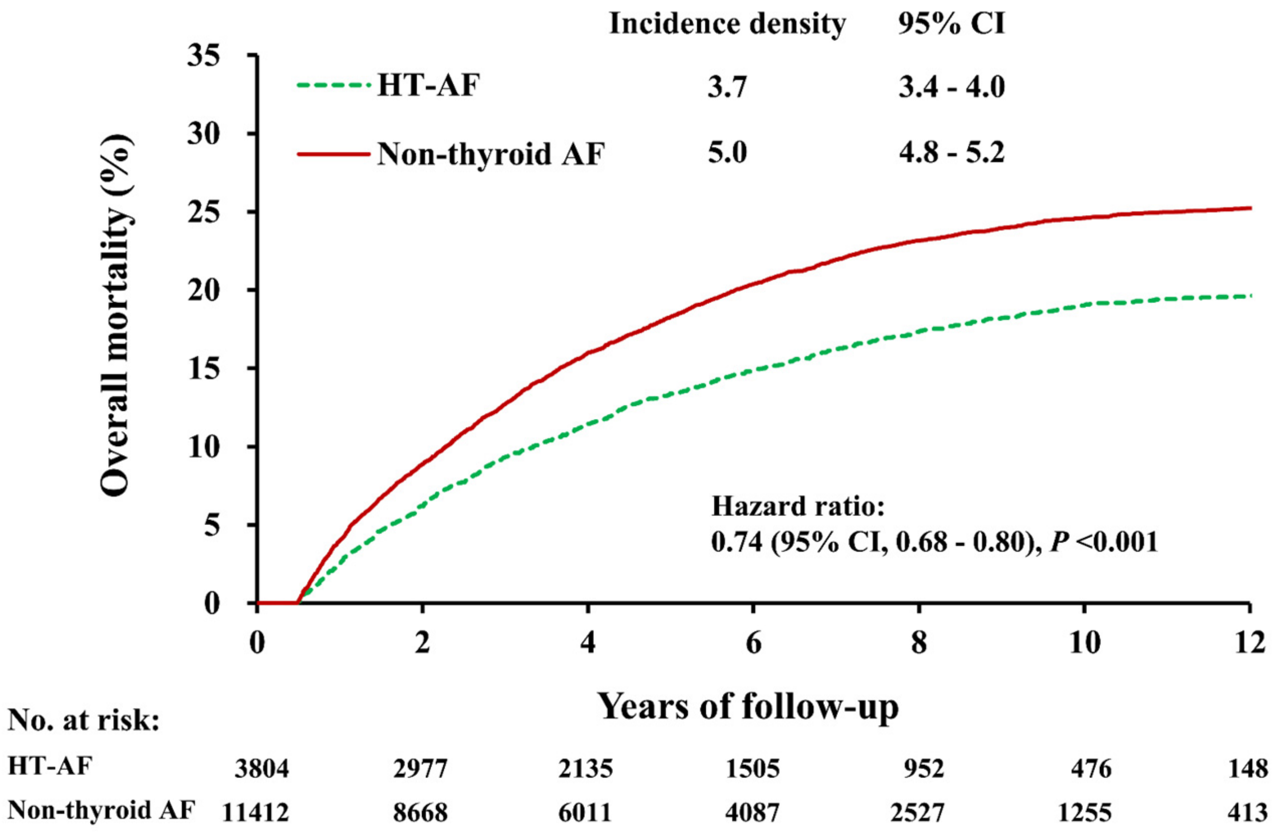

Figure S2. Cumulative event rates of ischemic stroke or systemic thromboembolism among patients who were prescribed different anti-thyroid drugs in the whole cohort (A), in those without anticoagulants (B) and in those with anticoagulants (C).

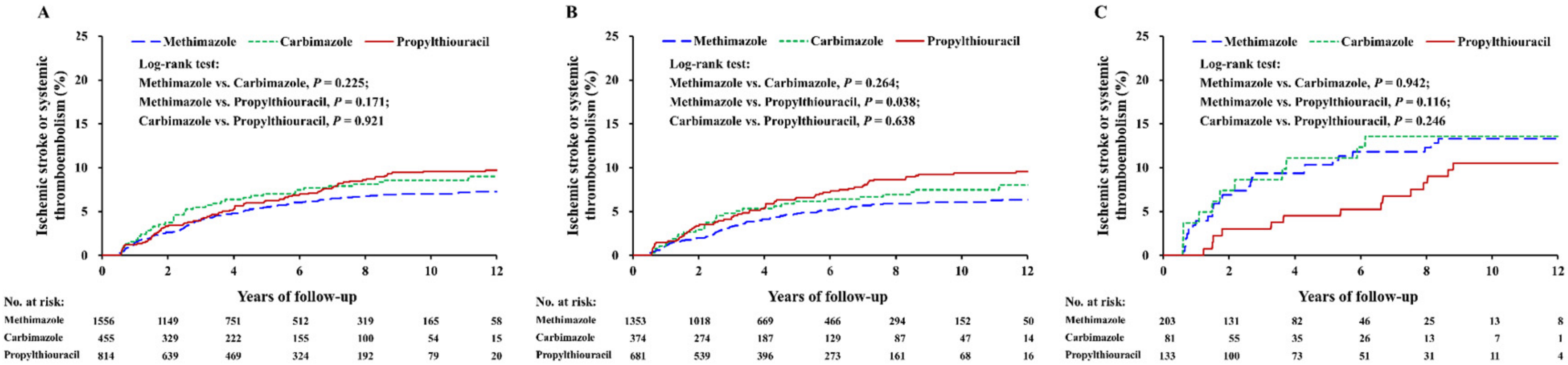

Supplement: Supplemental file. — Tables S1 & S2 and Figures S1 & S2. [file gh-16-1-871-s1.pdf]
